# Engineered Trx2p industrial yeast strain protects glycolysis and fermentation proteins from oxidative carbonylation during biomass propagation

**DOI:** 10.1186/1475-2859-11-4

**Published:** 2012-01-09

**Authors:** Rocío Gómez-Pastor, Roberto Pérez-Torrado, Elisa Cabiscol, Joaquim Ros, Emilia Matallana

**Affiliations:** 1 Departamento de Bioquímica y Biología Molecular, Universitat de València, Valencia, Spain; 2 Departamento de Biotecnología, Instituto de Agroquímica y Tecnología de Alimentos, CSIC, Apartado de Correos, 73. Burjassot (Valencia). E-46100, Spain; 3 Departament de Ciències Mèdiques Bàsiques, IRBLleida, Universitat de Lleida, Spain

**Keywords:** Thioredoxins, Carbonylation, Yeasts, Biomass, Stress

## Abstract

**Background:**

In the yeast biomass production process, protein carbonylation has severe adverse effects since it diminishes biomass yield and profitability of industrial production plants. However, this significant detriment of yeast performance can be alleviated by increasing thioredoxins levels. Thioredoxins are important antioxidant defenses implicated in many functions in cells, and their primordial functions include scavenging of reactive oxygen species that produce dramatic and irreversible alterations such as protein carbonylation.

**Results:**

In this work we have found several proteins specifically protected by yeast Thioredoxin 2 (Trx2p). Bidimensional electrophoresis and carbonylated protein identification from *TRX*-deficient and *TRX*-overexpressing cells revealed that glycolysis and fermentation-related proteins are specific targets of Trx2p protection. Indeed, the *TRX2 *overexpressing strain presented increased activity of the central carbon metabolism enzymes. Interestingly, Trx2p specifically preserved alcohol dehydrogenase I (Adh1p) from carbonylation, decreased oligomer aggregates and increased its enzymatic activity.

**Conclusions:**

The identified proteins suggest that the fermentative capacity detriment observed under industrial conditions in T73 wine commercial strain results from the oxidative carbonylation of specific glycolytic and fermentation enzymes. Indeed, increased thioredoxin levels enhance the performance of key fermentation enzymes such as Adh1p, which consequently increases fermentative capacity.

## Background

In the industrial yeast biomass propagation process, oxidative stress plays an important role by decreasing biomass yield and affecting fermentative properties of the produced biomass [1,2]. Studying oxidative stress during the biomass propagation process is essential to obtain stress-resistant yeasts that are able to complete the industrial process with no detriment of their fermentative and growth properties. Under industrial conditions, many cellular components are negatively affected since lipid peroxidation increases, while total glutathione and catalase activity decrease [3]. However, *TRX2 *gene overexpression improves *Saccharomyces cerevisiae *oxidative stress response by diminishing the damage caused by ROS (reactive oxygen species) accumulation [3]. Trx2p is part of the cytosolic TRX system (thioredoxin1, thioredoxin 2, TRX reductase and NADPH) which reduces oxidized cysteine groups on proteins [4]. Thioredoxins act as reducing agents of the oxidized form of TRX peroxidase (*TSA1*), then favoring the action of reduced TRX peroxidase which scavenges ROS such as H_2_O_2 _[4]. Thioredoxins are involved in many cellular processes as a result of their oxidoreductase activity. They participate in sulphate metabolism by reducing PAPS enzyme (3'-phosphoadenosine 5'-phosphosulphate reductase) [5] and maintaining the dNTP synthesis rate during the S-phase by acting as an electron donor of ribonucleotide reductase [6]. Thioredoxins are also involved in protein folding, regulation of transcription factors [7] and protein repair after oxidative damage [8].

Thioredoxins regulate hydrogen peroxide-induced signaling pathways in yeast, like inactivation of Yap1p, the main oxidative AP-1-like transcription factor [8]. TRXs have also been implicated in the regulation of the redox state of H_2_O_2_-responsive signaling proteins, and they have many growth factor-like properties, including secretion, cell surface binding and catalytic activity [9]. In *E. coli *and plants, approximately 80 proteins have been associated with thioredoxins under different conditions, implicating thioredoxins in at least 26 cellular processes [10,11].

Damage caused by ROS accumulation includes oxidative alteration of cell components such as proteins, DNA and lipids. Under optimal physiological conditions, oxidative damage is minimized by antioxidant defenses that scavenge or prevent the generation of ROS, and repair or degrade oxidized molecules [12]. However under several stressful conditions, *S. cerevisiae *accumulates ROS, which oxidizes methionine residues, lowers the GSH/GSSG ratio and generates protein carbonylation [13,14]. Accumulation of protein-carbonyls groups by ROS is an important oxidative damage since it has been described as an irreversible modification, and it may function as a marker of oxidative stress, aging and age-related diseases [15,16]. Oxidatively modified proteins are generally dysfunctional, lose catalytic activity or structural integrity, and increase protein aggregates. Indeed, excessive carbonylation can cause cross-linked protein aggregates that are unable to be degraded by proteasome [16,17]. On the other hand, there are other oxidative modifications that are not necessarily negative, like S-thiolation, which is a reversible oxidative modification protecting proteins from carbonylation [18].

Increased levels of carbonylated proteins during aging and in response to oxidative stress are not random, and some proteins are more susceptible than others, especially those with a transition metal-binding site in their catalytic center [19]. Besides, selectivity of protein carbonylation is clearly demonstrated by the fact that the relative amount of each specific protein is not a determining factor in the degree of carbonylation [14]. However, this phenomenon is not well understood because some proteins exhibit a high carbonylation threshold without their catalytic function being affected [20].

We have previously demonstrated that *TRX2 *overexpression is able to lower global carbonylation levels during bench top trials using *S. cerevisiae *industrial strains [3]. The molecular mechanism of protein protection from oxidative carbonylation by thioredoxins has not yet been demonstrated, although recent studies suggested that thioredoxins are implicated in the decarbonylation mechanisms of oxidized proteins [21]. Since cysteine residues are susceptible to being modified by carbonylation events [22], activity of thioredoxins as thiol-disulphide oxidoreductase and their participation in S-glutathionylation, the major form of S-thiolation [23], could play a key role in decreased carbonylation. Another suggested relationship between thioredoxins and oxidative carbonylation is the drop in Fe release from important proteins, like aconitase, as a result of diminished oxidative stress [24,25].

Other cellular antioxidant components, such as the methionine sulfoxide reductase A (MsrA), have been shown to be capable of preventing protein carbonylation in yeast and mice [26]. Moreover, there have been reports that calorie restriction lowers specific carbonylation in the proteins involved in glycolysis and chaperons by increasing superoxide dismutase and catalase activities and by extending lifespan [20]. Besides, experiments with yeast cells under aerobic conditions that are shifted to anoxia have revealed high carbonylation levels in glycolytic and mitochondrial proteins due to lack of oxygen [27]. This phenomenon could be associated with the transcription decrease of oxidative stress-related genes in anoxia, like the *TRX2 *gene [2].

In the present work, we have identified different proteins whose implication in essential biological processes for yeast growth is relevant and which are specifically affected by oxidative carbonylation during biomass propagation. This work also contributes to the identification of new targets of thioredoxins that are protected against carbonylation damage. One example is Adh1p, which presents lowered carbonylation and reduced oligomer aggregation in a *TRX2 *overexpressing strain.

## Results

### Oxidative damage increases in specific proteins during the biomass propagation process

**Figure 1:**
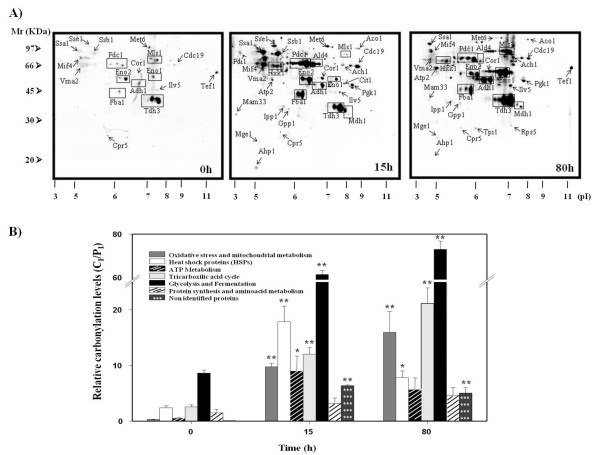
**Protein oxidation of T73 strain during biomass propagation**. (A) Anti-DNP western blots from 2D gels obtained from cells collected at 0 h, 15 h and 80 h of growth during industrial biomass propagation. A representative 2D-gel from triplicate experiments is shown. (B) Relative carbonylation levels of oxidized proteins grouped into different functional categories defined in Table 1. The ratio C_I _(carbonyl intensity)/P_I _(protein intensity) of each functional category was calculated as the summatory of C_I_/P_I _for each protein belonging to the respective functional category. Statistical comparisons with reference sample (Time 0 h) were made using a Student's *t-*test (* p < 0.05).

One of the most studied markers resulting from oxidative stress is protein carbonyl formation [14,28]. During wine yeast biomass propagation, cells are exposed to oxidative stress conditions at different critical time points [3,29]. Here we studied protein carbonylation during industrial propagation in yeast extracts by performing protein separation in 2D gels (additional file 1) and western blots anti DNP. Figure 1A provides three representative samples (0 h, 15 h and 80 h), showing increased protein damage during the process. At the starting time (0 h), just a few spots appeared to be carbonylated. During the diauxic shift (15 h), an increase in carbonylated spots was noted if compared to time 0 h. At the end of the process, the number of carbonylated spots was similar to that obtained at 15 h, but carbonyl intensity was greater. A total of 47 spots had been differentially carbonylated during biomass propagation and corresponded to 34 identified proteins (Table 1). Twenty-four proteins were identified by mass spectrometry and 10 proteins were identified by gel matching. The 34 identified proteins were grouped into six functional categories: glycolysis and fermentation, oxidative stress and mitochondrial metabolism, heat shock proteins, ATP metabolism, tricarboxylic acid cycle, and protein synthesis and amino acid metabolism. Figure 1B depicts the relative carbonylation levels for each functional category during industrial process. The most representative functional category was glycolysis and fermentation, which includes 12 specifically carbonylated proteins (Table 1). Key enzymes in fermentation processes such as Tdh3p, Eno1p and Adh1p were heavily damaged. The results show how the carbonylation of some proteins belonging to heat shock proteins (Mif4p and Ssa1p) and ATP metabolism (Vma1p and Atp2p) specifically increased at 15 h of growth, which lowered at the end of the process. On the other hand, the proteins grouped into oxidative stress and mitochondrial metabolism (Cor1p, Ilv5p), tricarboxylic acid cycle (Mls1p, Ach1p) and glycolysis and fermentation, presented significantly increased carbonylation levels at the end of the process.

**Table 1:** Carbonylated proteins during biomass propagation process for T73 wine yeast strain.

Protein name	Description	Molecular function	^a^C_I_/P_I_ Time 0 h	^a^C_I_/P_I_ Time 15 h	^a^C_I_/P_I_ Time 80 h
**Oxidative stress and mitochondrial metabolism**					
Ilv5p	Acetohydroxyacid reductoisomerase	Mitochondrial DNA stability	**0.16**	**2.53**	**10.20**
Cor1p	Ubiquinol-cytochrome c reductase (bc1 complex)	Electron transport chain	**0.03**	**2.28**	**3.89**
Pdi1p	Protein disulfide isomerase	Formation of disulfide bonds	**Nd**	**2.44**	**0.12**
Mam33p	Mitochondrial protein	Oxidative phosphorylation	**Nd**	**1.59**	**0.39**
Ahp1	Peroxiredoxin type II	Oxidative damage protection	**Nd**	**0.91**	**0.22**
**Heat shock proteins**					
Ssa1p	Heat shock protein 70	Protein folding	**1.01**	**2.45**	**1.49**
Mif4p	Heat shock protein 60	Mitochondrial protein folding	**0.65**	**8.37**	**3.77**
Ssb1p	Cytoplasmic ATPase	Protein folding	**0.36**	**4.78**	**1.44**
Sse1p	Heat shock protein 90	Binds unfolded proteins	**0.06**	**2.30**	**1.20**
Cpr5p	Peptidyl-prolyl-cis-trans isomerase	Response to unfolded proteins	**0.05**	**0.92**	**0.55**
Mge1p	Mitochondrial co-chaperone	Folding of Fe/S cluster proteins	**Nd**	**0.08**	**0.03**
**ATP Metabolism**					
Vma1p	Vacuolar H+ATPase	Vacuolar acidification	**0.40**	**4.08**	**0.71**
Ipp1p	Inorganic pirophosphatase	Exchange of O_2 _from Pi with water	**Nd**	**1.96**	**0.86**
ATp2p	ATP synthase (β-sub F1)	ATP synthesis	**Nd**	**2.95**	**4.08**
**Tricarboxilic acid cycle**					
Mls1p	Malate synthase	Enzyme of the glyoxylate cycle	**0.73**	**0.62**	**9.07**
Mdh1p	Malate dehydrogenase	Conversion malate and oxaloacetate	**Nd**	**2.23**	**1.03**
Cit1p	Citrate synthase	Condensation of acetyl coenzyme A and oxaloacetate to form citrate	**Nd**	**1.35**	**0.67**
Aco1p	Aconitase I	Tricarboxilic acid cycle	**Nd**	**1.23**	**1.36**
Ach1p	CoA transferase	Acetate utilization	**Nd**	**0.98**	**4.08**
**Glycolysis and Fermentation**					
Tdh3p	Glyceraldehyde-3-phosphate dehydrogenase	Glycolysis	**3.69**	**7.23**	**24.22**
Eno1p	Enolase I	Glycolysis	**1.17**	**2.86**	**11.12**
Adh1p	Alcohol dehydrogenase	Alcoholic fermentation	**0.94**	**4.67**	**8.32**
Pdc1p	Piruvate dehydrogenase	Alcoholic fermentation	**0.53**	**13.21**	**4.48**
Eno2p	Enolase II	Glycolysis and Gluconeogenesis	**0.34**	**7.67**	**6.25**
Fba1p	Fructose-1,6-bisphosphate aldolase	Glycolysis	**0.16**	**6.98**	**6.22**
Cdc19p	Piruvate kinase	Glycolysis	**0.04**	**1.89**	**1.50**
Ald4p	Aldehyde dehydrogenase	Required for growth on ethanol	**Nd**	**7.78**	**4.17**
Hxk1p	Hexokinase	Glucose biosynthesis	**Nd**	**4.37**	**2.79**
Gpp1p	DL-glycerol-3-phosphatase	Glycerol biosynthesis	**Nd**	**2.05**	**1.51**
Pgk1p	3-phosphoglycerate kinase	Glycolysis and Gluconeogenesis	**Nd**	**1.81**	**1.78**
Tpi1p	Triose phosphate isomerase	Glycolysis	**Nd**	**Nd**	**3.21**
**Protein synthesis and amino acid metabolism**					
Tef1p	Translational elongation factor EF-1a	Binding of AA-tRNA to ribosomes	**1.36**	**0.85**	**1.15**
Met6p	Methionine synthase	Methionine synthesis	**0.67**	**2.57**	**1.85**
Rps5p	Protein component of the 40S ribosome	Protein synthesis	**Nd**	**Nd**	**1.57**

**^a^: **Represented data correspond to C_I _(carbonyl intensity from western 2-D anti-DNP) normalized with P_I _(protein intensity) from 2-D gels (additional file 1). Underlined proteins were identified by mass spectrolmetry. **Nd**: no detected as carbonylated spots.

The obtained carbonylation profiles correlate with those previously published by one-dimensional anti-DNP western blot [3], where a group of proteins showed peaked carbonylation during the industrial propagation process, whereas other proteins presented an accumulation of carbonyl content at the end.

### *TRX2 *overexpression protects proteins against oxidative carbonylation

**Figure 2:**
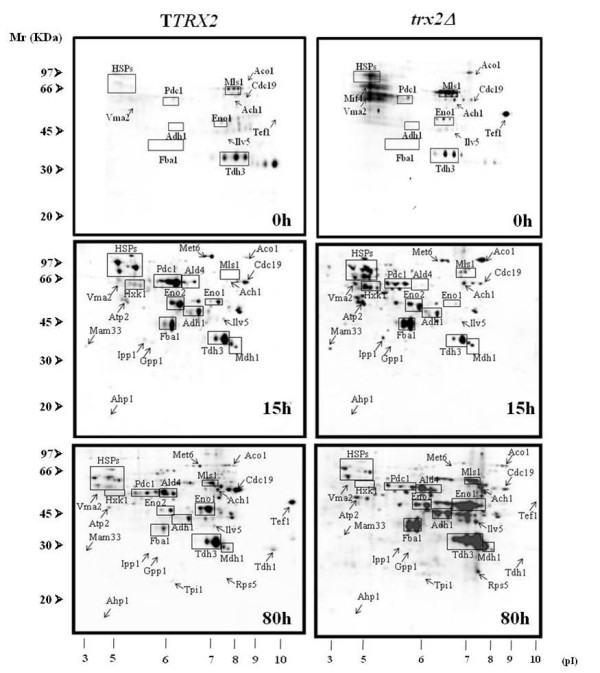
**Differential carbonylation profiles using *TRX2 *gene modified strains**. (A) Anti-DNP western blots from the 2D gels obtained from cells collected at 15 h and 80 h of growth under industrial conditions. Marked proteins present significant oxidative damage variation compared to the T73 control strain among the three biological replicates. (B) Silver-stained gels as a control of protein amount for each sample.

Little is known about the specific protein targets of thioredoxins in yeast. TRXs have been described to interact directly with Ahp1p, Tsa1p and Met16p when performing yeast two-hybrid knockout experiments [30]. However, their oxidoreductase activity can be extended to other protein not yet discovered. In order to identify possible targets in *S. cerevisiae*, we compared the oxidation level of specifically carbonylated proteins in the T73 control strain during industrial biomass propagation with the oxidation levels obtained for the *TRX2 *gene-modified strains. Trx2p target proteins display lower oxidation levels when Trx2p overaccumulates, and higher levels when it is lacking. Figure 2 illustrates the western blot anti-DNP of 2D gels from the T*TRX2 *and *trx2 *strains at 15 h and 80 h of growth during biomass propagation. The experiments were carried out in triplicate by performing 2-D gels in parallel with control strain T73. A total of 29 proteins were differentially carbonylated in both strains throughout the process (Table 2), and 22 proteins were identified by mass spectrometry. The relative carbonyl levels were analyzed as the ratio of carbonyl intensity of each protein and its corresponding protein intensity (additional file 2) when compared to the values obtained for the control strain. Ratio values of up to 1.5 fold increase or decrease if compared to the T73 strain were considered significant. At the starting point (time 0 h) just few proteins were differentially carbonylated in T*TRX2 *and *trx2 *strains comparing to control strain T73, where heat shock proteins showed a significant increase in carbonylation level for *trx2*. The T*TRX2 *strain showed 13 proteins with a significant carbonylation level decrease at 15 h of growth, which were grouped into the six defined functional categories. These proteins include Ahp1p, Sse1p, Vma1p and Ald4p as the most down-carbonylated proteins. At the end of the process, when cells had been under respiratory conditions for more than 40 h, we found 12 proteins with a significant carbonylation level decrease if compared to the control strain. The proteins with least oxidative damage were Hxk1p, Fba1p, Tdh3p and Adh1p, all of which are involved in glycolysis and fermentation, and were greatly damaged in the T73 strain.

**Table 2:** Differentially carbonylated proteins in TRX2 gene modified strains along biomass propagation process.

Protein name	Time 0 h		Time 15 h		Time 80 h	
	T*TRX2 *vs T73	trx2 vs T73	T*TRX2 *vs T73	Trx2 vs T73	T*TRX2 *vs T73	Trx2 vs T73
**Oxidative stress and mitochondrial metabolism**						
Ilv5*	Nv	Nv	0.52 (↓)	0.38 (↓↓)	**0.45 (**↓**)**	**Nv**
Ahp1p	Nv	Nv	0.23 (↓↓)	0.26 (↓↓)	Nv	Nv
**Heat shock proteins**						
Ssa1p	Nv	1.98 (↑)	0.75	0.52	0.75	Nv
Mif4p*	0.50 (↓)	2.93 (↑)	0.57 (↓)	0.49 (↓)	**0.36 (↓)**	**Nv**
Ssb1p*	**Nv**	**Nv**	**0.86**	**2.38 (↑)**	**1.43**	**2.37 (↑)**
Sse1p*	**0.81**	**1.23**	**0.22 (↓↓)**	**0.87**	1.38	Nv
Cpr5p	Nv	Nv	Nv	Nv	0.82	0.85
**ATP Metabolism**						
Vam1p*	**0.92**	**3.05 (↑↑)**	0.15 (↓↓)	0.54 (↓)	0.53 (↓)	0.69
Ipp1p*	**Nv**	**Nv**	**0.65**	**2.24 (↑)**	Nv	Nv
Atp2p*	**Nv**	**Nv**	**0.51 (↓)**	**3.86 (↑↑)**	**0.37 (↓)**	**Nv**
Mam33p*	**Nv**	**Nv**	**0.48 (↓)**	**2.13 (↑)**	**0.86**	**1.72 (↑)**
**Tricarboxilic acid cycle**						
Mls1p*	Nv	2.03 (↑)	**0.75**	**1.79 (↑)**	0.11 (↓↓)	0.88
Mdh1p*	Nv	Nv	1.68 (↑)	2.01 (↑)	Nv	2.67 (↑)
Aco1p*	**Nv**	**1.53 (↑)**	**0.86**	**4.03 (↑↑)**	**0.76**	**1.91 (↑)**
**Glycolysis and Fermentation**						
Tdh3p*	**0.81**	**0.75**	**0.71**	**0.62**	0.24 (↓↓)	2.38 ()
Eno1p*	**Nv**	**Nv**	**Nv**	**0.24 (↓↓)**	0.32 (↓)	3.61 (↑↑)
Adh1p*	**Nv**	**Nv**	**0.95**	**0.98**	**0.13 (↓↓)**	**Nv**
Pdc1p	**Nv**	**Nv**	0.56 (↓)	0.45 (↓)	0.65	0.64
Eno2p*	**0.46 (↓)**	**0.53 (↓)**	0.85	0.52 (↓)	0.29 (↓)	Nv
Fba1p*	**Nv**	**Nv**	**0.52 (↓)**	**2.27 (↑)**	**0.18 (↓↓)**	**4.07 (↑↑)**
Cdc19p*	**Nv**	**Nv**	0.79	0.60	2.76 (↑)	Nv
Adl4p	**Nv**	**Nv**	0.21 (↓↓)	0.15 (↓↓)	3.59 (↑↑)	4.28 (↑↑)
Hxk1p*	**Nv**	**Nv**	**0.33 (↓)**	**2.01 (↑)**	0.20 (↓↓)	0.27 (↓)
Gpp1p	Nv	Nv	0.53 (↓)	0.41 (↓)	0.85	Nv
Tpi1p	Nv	Nv	Nv	Nv	0.85	0.84
Tdh1p*	1.53 (↑)	Nv	Nv	Nv	1.84 (↑)	Nv
**Protein synthesis**						
Tef1p	0.28 (↓↓)	1.92 (↑)	Nv	Nv	**1.82 (↑)**	**0.12 (↓↓)**
Met6p	Nv	Nv	Nv	Nv	**Nv**	**0.85**
Rps5p*	Nv	Nv	Nv	Nv	**Nv**	**4.31 (↑↑)**

Represented data correspond to normalized Carbonyl intensity (C_I_) values from Western 2-D anti-DNP and Protein intensity (P_I_) from 2-D geles (Additional file 2) between the modified strain and control strains respectively. Ratios in bold character represent proteins with differential carbonylation pattern among T*TRX2 *and *trx2 *strains.

(↑) ratio [1.5-3]. (↑↑) ratio [ > 3], (↓) [0.5-0.25]. (↓↓) ratio [ < 0.25]

**Nv**: No variation respect to carbonylation levels in T73 control strain.

* Putative targets of Trx2p

Conversely, the *trx2 *strain showed 12 proteins with reduced oxidative damage at 15 h of growth, some of which were similar to those founded in the T*TRX2 *strain. The fact that we found some proteins which presented a similar oxidative damage decrease among both modified strains could be associated with a compensatory phenomenon resulting from the lack of the *TRX2 *gene. However, the *trx2 *strain presented 9 proteins which were more oxidized than the control strain, whereas Mam33p, Atp2p, Ipp1p, Aco1p, Hkx1p and Fba1p were less oxidized in T*TRX2*.

At 80 h of growth, the *trx2 *strain showed 8 proteins which presented a higher carbonylation ratio, and 3 proteins with low carbonylation levels. Among the more oxidized proteins we found Rps5p, Fba1p, Eno1p and Ald4p. Besides, Mam33p, Aco1p and Fba1p were more oxidized in the *trx2 *strain when compared to T*TRX2 *in both analyzed time samplings. In summary, the proteins with low oxidative damage levels in the T*TRX2 *strain or with high carbonylation levels in the *trx2 *strain at any time point were putative thioredoxin targets. Based on these criteria, 22 proteins (Aco1p, Adh1p, Atp2p, Cdc19p, Eno1p, Eno2p, Fba1p, Hxk1p, Ilv5p, Ipp1p, Mam33p, Mdh1p, Mif4p, Mls1p, Rsp5, Ssa1p, Ssb1p, Sse1p, Tdh1p, Tdh3p, Tpi1p, and Vma1p) were identified as presumable Trx2p-protected polypeptides.

Additional file 3 provides the different carbonylation profiles for each functional category at 15 h and 80 h of growth in the three T73, T*TRX2 *and *trx2 *strains. The relative carbonylation levels of oxidative stress-related proteins presented significantly increased oxidative damage at the end of the process in the T73 and *trx2 *strains (additional file 3 panel A). On the other hand, the T*TRX2 *strain displayed low carbonylation levels at both time points. Similar results were obtained for the proteins grouped in the functional tricarboxylic acid cycle (additional file 3 panel D) and glycolysis and fermentation categories (additional file 3 panel E). For the heat shock proteins (HSPs) (additional file 3 panel B), we observed a high oxidative damage level at the starting time of the process in T73 and *trx2*, whereas it was lower for T*TRX2*. HSPs had reduced protein-carbonyls content at the end of the process in all the strains. However, T*TRX2 *presented once again the lowest carbonylation level. Regarding the proteins related to ATP metabolism (additional file 3 panel C), the *trx2 *strain showed the highest oxidation levels at 15 h of growth. In contrast, T*TRX2 *had the lowest carbonylation levels at both time points. Finally, the proteins related to protein synthesis and amino acid metabolism (additional file 3 panel F) had similar carbonylation profiles in all the strains, except for an increase noted in the *trx2 *strain at 80 h.

### Oxidative carbonylation lowers glycolytic enzyme activities

**Figure 3:**
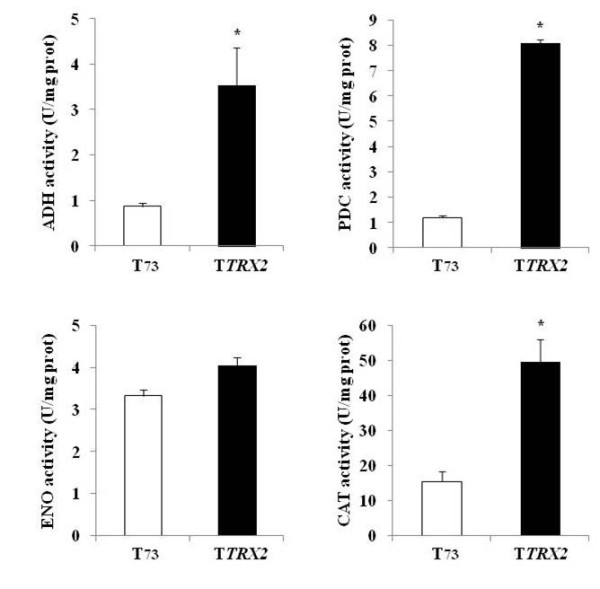
**Enzyme activities of oxidative damaged proteins**. The activities of ADH (alcohol dehydrogenase), PDC (pyruvate decarboxylase), ENO (enolase) and CAT (catalase) were determined as described in Materials and Methods for the T73 and T*TRX2 *strains. All the data are expressed as means ± standard deviation of three biological replicates. Statistical comparisons between T73 and T*TRX2 *strains were made using a Student's *t-*test (* p < 0.05).

To check whether the increased carbonyl content in glycolytic proteins could affect their corresponding enzymatic function after industrial propagation, we analyzed the enzyme activities of ADH, PDC and ENO in the T73 and T*TRX2 *strains. Catalase activity (CAT) was also assayed because it is a good marker of lowered enzyme activity when it undergoes oxidative carbonylation [20]. We inoculated the active dry biomass obtained at the end of the propagation process in YPGF medium to simulate fermentation conditions. Figure 3 shows the enzyme activity of ADH, PDC, ENO and CAT for the T73 and T*TRX2 *strains. T*TRX2 *displayed significantly higher ADH, PDC and CAT activities than the control strain correlating with the low carbonylation levels observed for the assayed enzymes. In correlation with these results, ENO and CAT activities decreased in *trx2 *1.35 fold and 2.15 fold respectively (data not shown). Despite the large differences observed for Eno1p and Eno2p carbonyl content in T*TRX2 *compared to the control strain, no significant differences were observed in ENO enzymatic activity.

### *TRX2 *gene overexpression prevents Adh1 protein carbonylation and aggregates formation

Adh1p, one of the most important enzymes in alcoholic fermentation, functions as a tetramer of four identical subunits of approximately 37 kDa containing two zinc ions each. Adh1p was one of the most carbonylated proteins among glycolytic enzymes in T73 during biomass propagation experiments (Table 1). Besides, T*TRX2 *significantly reduced Adh1p protein oxidation and it was the least damaged protein compared to the control strain at the end of the process (Table 2). Thus, we wanted to study the Adh1p levels in the biomass production process but also in active dry yeast and in the same biomass inoculated in YPGF medium, where alcoholic fermentation occurs and Adh1p might have the most important role for the cell. We first analyzed global protein carbonylation under these conditions (additional file 4) and we observed that drying and YPGF re-inoculation also increased carbonyl levels after biomass propagation in T73 (1.71 fold and 2.35 fold respectively) whereas T*TRX2 *strain displayed lower carbonyl levels in all conditions (1.35 fold and 1.71 fold respectively). The reason for using cells in YPGF after 5 h of growth is to check if oxidized protein level changes under new fermentation conditions. However, we observed an additional increase in protein oxidation in YPGF medium especially for the control strain (additional file 4), which also correlated with the previous differences observed for fermentative capacity under these conditions between T73 and T*TRX2 *strains [31].

**Figure 4:**
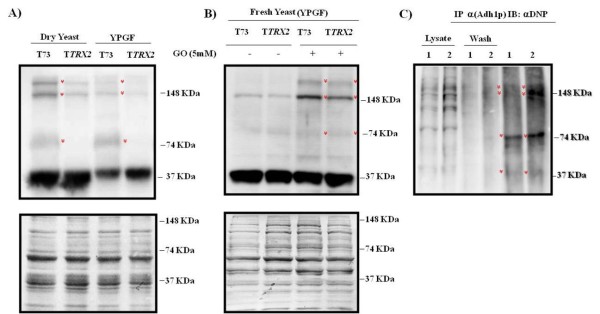
**Adh1p aggregation resulting from oxidative carbonylation**. Anti-Adh1p western blot (upper panel) for the T73 and T*TRX2 *strains from dehydrated cells and cells grown in YPGF medium for 5 h (A) or from fresh YPGF growing cells, treated or not treated with carbonylation inductor glyoxal (GO) (B). The bottom panel shows the Coomassie-stained membrane used as a control of protein amount. (C) Anti-DNP western blot of Adh1p immunoprecipitated samples: ^1 ^T73 and ^2 ^T73+5 mM glyoxal. Marked bands correspond to the dimer (74 KDa) and tetramer (148 KDa) forms of Adh1p.

Figure 4 depicts the anti-Adh1p western blot experiments for the T73 and T*TRX2 *strains. The upper panel 4 A shows the anti-Adh1p western blot where three different bands can be observed in the T73 strain, corresponding to the monomer (37 kDa), dimer (74 kDa) and tetramer (148 kDa) forms of Adh1p, and one additional band higher than 148 kDa, despite the western blot being performed under denaturing conditions. Unlike the control strain, *TRX2 *overexpressing cells showed Adh1p mainly in the monomer form. In addition, the amount of Adh1p monomer form in the T73 strain significantly decreased after growing in YPGF compared to dry yeast. However, the dimer and tetramer forms were still present under these conditions, suggesting an Adh1p monomer turnover due to its high oxidation level, whereas the other forms were not degraded. For the T*TRX2 *strain, the Adh1p amount in YPGF slightly lowered, and neither the dimer nor the tetramer forms were observed.

In order to confirm that the different bands observed for Adh1p in the T73 strain are due to protein aggregates caused by carbonylation damage, we inoculated YPGF medium with fresh cells or cells treated with the carbonylation inductor glyoxal (GO) [32]. In Figure 4B we can observe for both strains that fresh yeast without treatment mainly showed the monomer form and very slight bands corresponding to the dimer and tetramer forms. After treatment with GO, the band corresponding to the tetramer (148 kDa) had increased and high weight forms appeared. Even under these induced conditions, the T*TRX2 *strain presented lower levels of tetrameric and high weight forms. To confirm that Adh1p aggregates were in fact carbonylated, Adh1p was immunoprecipitated and a subsequent western blot to detect carbonylation was performed (Figure 4C). The results confirm that high molecular weight Adh1p aggregates, corresponding to the size of dimers (74 kDa), tetramers (148 kDa), and higher oligomers, are indeed carbonylated. These results indicate that *TRX2 *overexpression increases Adh1p monomer stability by decreasing protein carbonylation and aggregates formation, thus maintaining its cellular concentration and increasing its enzyme activity.

## Discussion

In this work, we have found that several proteins implicated mainly in glycolysis and fermentation become specifically carbonylated throughout industrial yeast biomass propagation, which affects their corresponding enzyme activities and lowers biomass fermentative capacity. This phenomenon can be directly related with the high oxidative stress which occurs at critical time points during the industrial process [2,3]. Under these conditions, we have previously observed improved biomass yield and fermentative capacity as a result of *TRX2 *gene overexpression [3]. We have demonstrated that *TRX2 *gene overexpression is able to reduce oxidative damages, global protein carbonylation and lipid peroxidation levels, and to increase total glutathione and antioxidant enzyme activities. The aim of the present study was to identify those proteins that present increased carbonylation after industrial biomass propagation and to find putative Trx2p protection targets that could highlight the improved phenotype of the T*TRX2 *strain [3].

From the western blot anti-DNP of 2D gels experiments using the T73 strain, we were able to identify selective protein carbonylation during the biomass propagation process. The main heat shock proteins Mif4p (Hsp60p), Ssa1p (Hsp70p) and Sse1p (Hsp90) become specifically carbonylated during the diauxic shift (15 h), as do the proteins relating with ATP metabolism. We obtained similar results for the oxidative stress-induced experiments by H_2_O_2 _[14] and by using chronologically aged cells [20]. These results suggest that oxidation of molecular chaperones may be due to their role as a proteins shield for ROS generated during the diauxic shift. Besides, another interesting oxidatively damaged protein at 15 h of growth is thioredoxin peroxidase Ahp1p, which uses thioredoxins as an electron donor and is highly implicated in the oxidative stress response [33]. By the end of the process, the carbonyl content of them all had lowered, but their protein levels either increased or did not vary [29]. Since carbonylation has been described as an irreversible process [15], the lower carbonyl content of these proteins can be explained by an effect of culture dilution as a consequence of biomass growth.

At the end of the propagation process, we observed that several proteins had been greatly damaged if compared to the initial growth steps. In this case, the proteins involved in glycolysis and fermentation, mitochondrial proteins (Cor5p and Ilv5p), and the proteins related to tricarboxylic acid cycle (Aco1p, Mls1p and Ach1p), all showed high carbonylation levels. We have previously demonstrated that cells grown under respiratory conditions (YPG medium) are better prepared to cope with oxidative stress than those grown under fermenting conditions (YPD medium), by reducing protein carbonyl content [34]. However, this work reveals that glycolytic enzymes undergo vast damage in the cells grown under respiratory conditions for a long period (40 h). Furthermore, there have been reports that prolonged cultivation of *S. cerevisiae *in aerobic, glucose-limited chemostat cultures progressively lowers glycolytic enzyme activities [35]. Among the most carbonylated glycolytic enzymes, we found Tdh3p (GAPDH), Eno1p and Adh1p, which are key enzymes in alcoholic fermentation. Two of them (Tdh3p and Adh1p) catalyze oxidative reactions by using NADH. These enzymes' high carbonylation levels could be the main reason to explain the fermentative capacity detriment previously observed in the T73 strain [2,3,36]. In fact, GAPDH has been identified as a target of oxidative modification in many different cellular systems, which suggests its possible regulatory role as a sensor of oxidative stress conditions [37]. Krobitsch and colleagues [38] provided the first direct evidence for the oxidative inhibition of Tdh3p, and other glycolytic enzymes [39], in a controlled response that enables cells to redirect their carbohydrate flux from glycolysis to the pentose phosphate pathway, generating NADPH under stressful conditions. In addition, we have previously shown that *ADH1 *gene overexpression increases the NAD^+^/NADH ratio, and increases chronological and replicative lifespan extension in *S. cerevisiae *by diminishing oxidative stress [40]. Thus, the Adh1p oxidative carbonylation and lowered ADH activity observed in the yeast propagation process may contribute to unbalance the NAD^+^/NADH ratio by enhancing oxidative stress.

It is interesting to note that most of the damaged proteins identified in our experiments undergo other oxidative modifications, especially at their cysteine residues [18]. Le Moan and colleagues [41] demonstrated that after H_2_O_2 _addition, the proteome of oxidized protein thiols of several stress chaperons and glycolytic enzymes depends exclusively on the thioredoxin functions controlling the activity of oxidized proteins. Besides, many of these proteins are also targets of S-glutathionylation, even in algae and mammal cells, suggesting an important role of the redox state in the regulation of the protein function for several organisms [42,43].

The *TRX2-*overexpressing strain displays lower carbonylation levels for the majority of damaged proteins observed in wild-type strain, especially at the end of the industrial process. Most of these proteins also present cysteine residues close to the active site or important Cys disulfide bonds for their structure and catalytic function [41]. The increased Trx2p dosage (additional file 5) seems to stabilize the redox state of these cysteine residues by avoiding oxidative damage. As a result of less protein oxidative damage in the main glycolytic enzymes, T*TRX2 *strain exhibits greater GAPDH and ADH enzyme activities, suggesting a direct correlation between low carbonylation levels and great enzyme activity. The fact that the T*TRX2 *strain exhibits high levels of total glutathione during biomass propagation [3], and that several important proteins have lower carbonyl content, suggests that S-glutathionylation could protect against carbonylation. In addition, Tdh3p and Adh1p are susceptible to being modified by S-thiolation under H_2_O_2 _induced oxidative stress, which diminishes enzyme activity by at least 70% [18]. Besides, it has been described that thioredoxins catalyze deglutathionylation in yeast by playing a key role in regulating the modification of proteins by the glutathione system [23]. The fact that the T*TRX2 *strain shows greater ADH and PDC activity after dehydration may also be associated with the fact that, under fermentative conditions, the deglutathionylation of both enzymes is higher than in the control strain as a result of *TRX2 *overexpression.

The improved fermentative capacity previously observed for the T*TRX2 *strain may be chiefly due to less oxidative damage in the aforementioned enzymes. In addition, increased alcohol dehydrogenase activity could also help reduce oxidative stress in this strain, and may be a reason for biomass yield improvement since *ADH1 *gene overexpression increases yeast replicative and chronological lifespan [3,40].

The comparison made between the T*TRX2 *and *trx2 *strains allowed us to identify new targets of Trx2p protection. Most of these proteins are related to mitochondria, even Fba1p, which is involved in glycolysis, but localizes to the mitochondrial surface upon oxidative stress [44]. This suggests an implication of cytosolic thioredoxins in mitochondrial protein protection [45] under high endogenous oxidative stress conditions, as in the biomass production process.

One remarkable finding from this work is that Trx2p overexpression reduces Adh1p oligomers by the crosslinking caused by oxidative carbonylation. Although mild oxidation of a protein increases its degradation by proteasome 20S [45], excessive oxidation and cross-linking of proteins render them resistant to proteolytic degradation by the proteasome [18]. In *E. coli*, more than 95% of total carbonylated proteins are insoluble proteins mainly detectable in an aggregate state [46]. These authors proposed that some carbonylated proteins escape degradation *in vivo *by forming carbonylated protein aggregates, thus becoming non degradable which contributes to senescence. In several human neurodegenerative diseases it has been described how the proteins related to glycolysis and energy metabolism, cytoskeleton, chaperoning, cellular stress responses and members of the ubiquitin-proteasome system become aggregated as a result of oxidative damage [47]. According to these data, we suggest that Trx2p is able to diminish protein carbonylation damage, thus preventing protein aggregates and oxidative damage expansion. The molecular mechanisms of Trx2p protein protection against oxidative carbonylation could be associated with other oxidative modifications, especially at protein cysteine residues, and this phenomenon deserves further investigation.

## Conclusions

In the present work, we have identified several proteins that are affected by oxidative carbonylation during yeast biomass propagation which are of relevant interest in fermentation processes. The identified proteins allowed us to conclude that oxidative carbonylation of the main glycolytic and fermentation enzymes is the most important reason for the fermentative capacity detriment observed under industrial conditions. Furthermore, higher thioredoxin levels enhance the performance of key fermentation enzymes such as Adh1p, and consequently increases fermentative capacity. Since the produced biomass needs to be of good quality, the results obtained for the T*TRX2 *strain suggest that improvement in the oxidative stress response during biomass propagation process is a good target for genetic and technologic manipulation.

## Materials and Methods

### Strains, media and cultivation conditions

We used *S. cerevisiae *strain T73 (CECT 1894) isolated from Alicante (Spain) musts [48], which is commercialized by Lallemand Inc. (Montreal, Canada). This strain has been widely used in several studies and has proven to be a good wine yeast model. This strain was previously genetically modified to T73ura3 [49] to construct other strains given the absence of auxotrophies in wine natural yeasts. These strains are also aneuploids, and have a chromosome number that is not a multiple of the haploid number, and they require several rounds of transformation for deletion gene construction.

The YEp-TRX2 plasmid was obtained by subcloning a 0.7 kb *Eco*RI fragment containing the yeast *TRX2 *gene and promoter in the episomal yeast plasmid Yep352 carrying selectable marker *URA3*. The T*TRX2 *strain [2] is a genetically-modified T73*ura3 *strain following the lithium acetate procedure as modified by [50].

Strain *trx2 *was obtained by sequential deletion of the two copies of the *TRX2 *gene in strain T73*ura3*. Disruption was carried out by homologous recombination at both ends of the *TRX2 *open reading frame of an integration cassette carrying a *kanR *marker gene flanked by *loxP *sites. Excision of the marker is inducible by the expression of Cre recombinase introduced into the same strain [51] to allow repeated disruptions. Integration of the cassette at the *TRX2 *locus and further excision of the *kanR *marker were confirmed by PCR analysis. The absence of any *TRX2 *gene product was confirmed by northern and western blot analyses. Uracil prototrophy was restored by introducing a 1.1-kb *Hind*III linear fragment containing the *URA3 *gene.

Precultures for industrial biomass propagation experiments were prepared in YPD liquid medium (1% Yeast extract, 2% Peptone, 2% Glucose) and were incubated at 30°C with shaking (250 rpm) for 12 h.

Molasses medium (diluted to 60 g of sucrose L^-1 ^for the batch phase or 100 g of sucrose L^-1 ^for the fed-batch phase) was supplemented with 7.5 g L^-1 ^of (NH_4_)_2_SO_4_, 3.5 g L^-1^of KH_2_PO_4_, 0.75 g L^-1 ^of MgSO_4_7H_2_O, 10 ml L^-1^of vitamin solution, and 1 ml L^-1 ^of antifoam 204 (Sigma, St. Louis, Mo.). Molasses and mineral solutions were autoclaved separately. The vitamin solution containing 50 mg L^-1 ^of D-biotin, 1 g L^-1 ^of calcium pantothenate, and 1 g L^-1 ^of thiamine hydrochloride was filter sterilized (0.2-μm pore size) prior to use in the molasses medium.

The liquid medium YPGF (1% Yeast extract, 2% Peptone, 10% Glucose, 10% Fructose) was used to inoculate fresh yeast and active dry yeast produced under industrial conditions to simulate must sugar content and wine fermentation conditions. YPGF medium was also supplemented with the carbonylation inductor glyoxal (GO) at 5 mM for 1 h to induce protein carbonylation damage [32].

### Industrial production conditions

Biomass propagation experiments were designed with two growth stages, batch and fed-batch, in a BIOFLO III bioreactor (NBS, New Jersey), and the technical parameters (agitation, pH and feed rate) were established as previously described [1,3,29]. Overnight YPD precultures were incubated at 30°C with shaking (250 rpm) (Time 0 h). The bioreactor containing 2 L sterilized molasses medium at pH 4.5 was then inoculated to an initial OD_600 nm _of 0.05. In the batch phase, cells consumed all the sucrose present in the medium using a fermentative metabolism. When sucrose was exhausted (12-15 h), cells changed their metabolism to respiration, allowing the consumption of the produced ethanol until approximately 40 h of the process. When ethanol was exhausted, the fed-batch phase started by feeding the reactor continuously with molasses medium at the desired flow rate until approximately 80 h, thus avoiding fermentative metabolism in order to gain the highest biomass yield. Three independent production experiments were carried out for the T73, T*TRX2 *and *trx2 *strains.

### Biomass drying and rehydration

At the end of the fed-batch fermentation, biomass was separated by centrifugation from the fermented media and subjected to several washing steps with distilled water. Concentrated biomass (500 mg) was collected in petri plates. Yeast biomass was dehydrated under air flux in a convection oven at 30°C until approximately 8% relative humidity (approximately 24 h) with opened petri plates [3]. Dehydrated biomass was collected in plastic bags and stored under vacuum conditions at room temperature during one week. Rehydration was performed in distilled water at 37°C during 10 min under static conditions and 10 min with shaking at 130 rpm [52].

### Protein extraction and two-dimensional gel electrophoresis

Cell samples (25 mg) were collected at 0 h, 15 h and 80 h of growth for protein extraction. Cells were resuspended in 150 μL extraction buffer (8 M Urea, 25 mM Tris-HCl pH 8.0), a mixture of protease inhibitors (200 μM phenylmethylsulphonyl fluoride (PMSF), 20 μM TPcK, 200 μM pepstatin A) and 0.2 g of glass beads. Cells were broken in Fast Prep (MP Bio) at 5.0 m/s for 45 sec on 3 occasions. After centrifugation at 12000 rpm for 10 min, the supernatant was sonicated and centrifuged again at 12000 rpm for 10 min. The protein concentration was determined with a Nanodrop ND-1000 UV/Vis spectrophotometer. Among 50-80 μg of protein were diluted in 340 μl of Rehydration Buffer (8 M Urea, 4% CHAPS (w/v), 50 mM DTT and 0.5% ampholytes (v/v) pH 3-10 (Amersham)). Isoelectric focusing (50-100 μg of protein) was performed in immobilised pH gradient strips (3-11 NL; Amersham). After the first dimension, strips were incubated for 20 min with 5 ml of a solution containing 10 mM 2,4-dinitrophenylhydrazine (DNPH) in 10% trifluoroacetic acid (TFA). This compound reacts with carbonyl groups in proteins. To stop this reaction, the strip was transferred to a 5 ml solution containing 0.4 M Tris, 6 M Urea, 2% SDS and 20% glycerol. Second dimension SDS-PAGE was performed on 18 × 18 cm 11% polyacrylamide gels.

Four strips were run in parallel, two strips for the wild type and two strips for the respective mutant strain each time. Gels were either transferred to PVDF membranes for western blot analysis or silver strained and scanned in a GS800 densitometer (Bio-Rad). In both cases, obtained images were analyzed with the PDQuest software (Bio-Rad). Gels were silver-stained using the PlusOne silver staining kit of General Electric Healthcare. PVDF membranes were silver-stained to control the protein load as described elsewhere [53].

### Western blot analysis and carbonyl content quantitation

Crude extracts were separated in 10% SDS-polyacrylamide gels and transferred to the PVDF membrane from one-dimensional gels. A polyclonal anti-Adh1p antibody was purchased from Acris (R1049) which is able to detect monomer, dimer and tetramer forms of Adh1p under SDS/PAGE conditions as described on the web site http://www.acris-antibodies.com/antibodies/r1049.htm and diluted to 1:1000. The Western blots from two-dimensional gels were prepared as described in the previous section. A 1:5000 dilution of antibody against DNP (Dako) was used. In both cases, a peroxidase-conjugated anti-rabbit antibody was used for detection. Images were acquired in a ChemiDoc XRS System (Bio-Rad) and analyzed with the Quantity One software (Bio-Rad).

Carbonyl content was quantified using the PDQuest software (Bio-Rad). Carbonylation levels for each protein are calculated by dividing the carbonyl intensity from western 2-D anti DNP (C_I_) by protein intensity (P_I_) from 2-D silver-stained gels.

### Protein identification by tryptic digestion and MALDI-TOF

Protein spots were excised from gels and subjected to *in situ *digestion with trypsin on a ZipPlate (Millipore). Gel pieces were washed with 25 mM ammonium bicarbonate and dehydrated with acetonitrile followed by (i) reduction of cysteines with 10 mM DTT, (ii) alkylation of free cysteines with 55 mM iodoacetamide, and (iii) *in situ *digestion with 170 ng of trypsin overnight at 30°C. Peptide extractions and washes were performed on a ZipPlate following the manufacturer's recommendations. Tryptic peptides were recovered in 5 ml of 0.1% TFA, 50% acetonitrile, and spotted onto a MALDI plate in the presence of a-ciano-4-hydroxycynamic acid. Spectra were obtained in an Applied Biosystems voyager DE PRO MALDI-TOF apparatus operating in the reflector mode. The spectra with higher resolutions than 8000 were obtained. External calibration was performed with calibration mixtures from Applied Biosystems. The acquired spectra were processed by Data Explorer (version 4.0). Proteins were identified by peptide mass fingerprinting searching against the Swiss-Prot database using MASCOT. Protein coverage for each spot in the MASCOT analysis of up to 50 percent was considered significant. Alternatively, proteins were identified by gel matching with *S. cerevisiae *two-dimensional gel electrophoresis maps available in the following databases: YPD (Yeast Proteome Database; http://www.proteome.com); YMP (Yeast Mitochondrial Proteome; http://www.biochem.oulu.fi/proteomics/ymp.html); and 2-DE *S. cerevisiae *(IPG6-12) http://www.weihenstephan.de/proteomik/. Functional group analysis was performed using the GOstat and GO term finder (SGD database) online applications with a false discovery rate of 5%. The represented data correspond to the average of three biological replicates.

### ADH immunoprecipitation

Cells in IP buffer (50 mM Tris-HCl, pH 7.5, 150 mM NaCl, 5 mM EDTA, 10% glycerol, 0.1% Nonidet-P40) plus 1X protease inhibitors (Roche) and PMSF 0.4 mM were broken with 0.5 g of glass beads in a Fast Prep (MP Bio) at 5.0 m/s for 45 s, 3 times. Lysates were clarified by centrifugation for 15 min at 14.000 rpm and added to 20 μl of Protein A/G PLUS-Agarose beads (Santa Cruz Biotechnology, INC) with bound polyclonal anti-ADH antibody (Acris antibodies). After a 4 h binding step at 4°C, beads were washed three times with IP buffer plus 0.5% TritonX-100 and 0.5 mM NaCl and resuspended in carbonyl derivatization buffer (25 mM Imidazol, 2 mM EDTA, pH 7.0, 6% SDS, 1X protease inhibitor) and boiled 3 min. Sample proteins were derivatized with DNPH and separated in a 9% acrylamide gel (BioRad Laboratories) and incubated with anti-DNP antibody at 1/3500 and anti-rabbit (1/5000).

### Enzyme activities

Dehydrated cells were inoculated (10^7 ^cells/mL) in YPGF and incubated at 30°C and 65 rpm for 5 h. Samples were collected at the end of incubation, and cell extracts were prepared using glass beads and assayed as described in the following references: alcohol dehydrogenase [54], enolase [55], pyruvate decarboxylase [56] and catalase [57].

## Abbreviations

ADH: Alcohol dehydrogenase; CAT: catalase; DNPH: 2,4-dinitrophenylhydrazine; DNP: dinitrophenol; ENO: enolase; GSH: Reduced glutathione form; GSSG: Oxidized glutathione form; HRP: Horseradish peroxidase; OD: Optical density; PBS: Phosphate buffered saline; PDC: Pyruvate decarboxylase; PMSF: Phenylmethylsulfonyl fluoride; ROS: Radical oxygen species; SOD: Superoxide dismutase; TCA: Trichloroacetic acid; TPcK: N-tosyl-L-phenylalanylchloromethyl ketone dinitrophenilhydrazine.

## Competing interests

The authors declare that they have no competing interests.

## Authors' contributions

RGP carried out most of the experiments and drafted the manuscript. RPT initiated the project, assisted with conception, data interpretation, statistical analyses and contributed to the writing of the manuscript. EC and JR assisted with the experimental design of the oxidative stress experiments. EM conceived the study, participated in its design, and contributed to the writing of the manuscript. All the authors have read and approved the final manuscript.

## Supplementary Material

Additional file 1 **Two dimensional protein gels during biomass propagation at 0 h, 15 h and 80 h for control strain T73**. Proteins were visualized with silver staining. The proteins whose intensity varied significantly (P < 0.05) among the three replicates for each time point were annotated on the gel. Click here for file

Additional file 2 **Two dimensional protein gels during biomass propagation at 0 h, 15 h and 80 h for *TRX2 *gene modified strains**. Proteins were visualized with silver staining and gels were used as loading control. The proteins whose intensity varied significantly (P < 0.05) among the three replicates for each time point were annotated on the gel. Click here for file

Additional file 3 **Carbonylation profile of the different protein functional categories during biomass propagation process**. Relative carbonyl content measured as C_I_/P_I _of each defined functional category at 15 h and 80 h of growth among the three strains T73, T*TRX2 *and *trx2*. All the data are expressed as means ± standard deviation. Comparisons among multiple groups were performed using the ANOVA (*a *is significantly different (p < 0.05) to *b *and significantly different to *c *(p < 0.05). Click here for file

Additional file 4 **Carbonyls levels after drying process and YPGF re-inocculation**. (A) anti-DNP monodimensional western blot at different time points of the industrial process and after drying and re-inocculation in YPGF for T73 and T*TRX2 *strains. (B) Relative carbonyl levels were quantified by using the QuantityOne software (Bio-Rad) and carbonylation levels were normalized with the protein amount. Click here for file

Additional file 5 **Trx2p visualization during biomass propagation**. (A). Magnified regions of the two-dimensional gels where Trx2p can be observed in the different strains (A) T73 and (B) T*TRX2 *at 15 h of the biomass propagation process. (B) Western blot anti-Trx2p during biomass propagation. Coomassie-stained membranes are shown as a control of protein amount. Click here for file
