# Supplementary figures and images for: Engineered Trx2p industrial yeast strain protects glycolysis and fermentation proteins from oxidative carbonylation during biomass propagation
Source: Microb Cell Fact. 2012 Jan 9;11:4. doi: 10.1186/1475-2859-11-4 (PMC3280929; doi:10.1186/1475-2859-11-4)

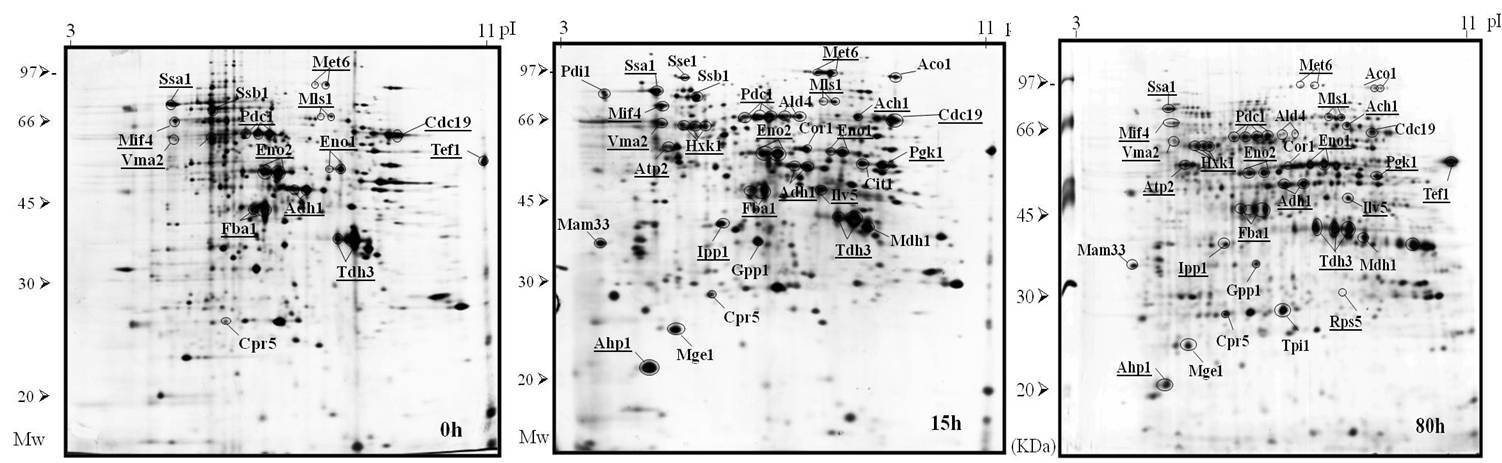

Supplement: Additional file 1 — Two dimensional protein gels during biomass propagation at 0 h, 15 h and 80 h for control strain T73. Proteins were visualized with silver staining. The proteins whose intensity varied significantly (P < 0.05) among the three replicates for each time point were annotated on the gel. [file 1475-2859-11-4-S1.JPEG]

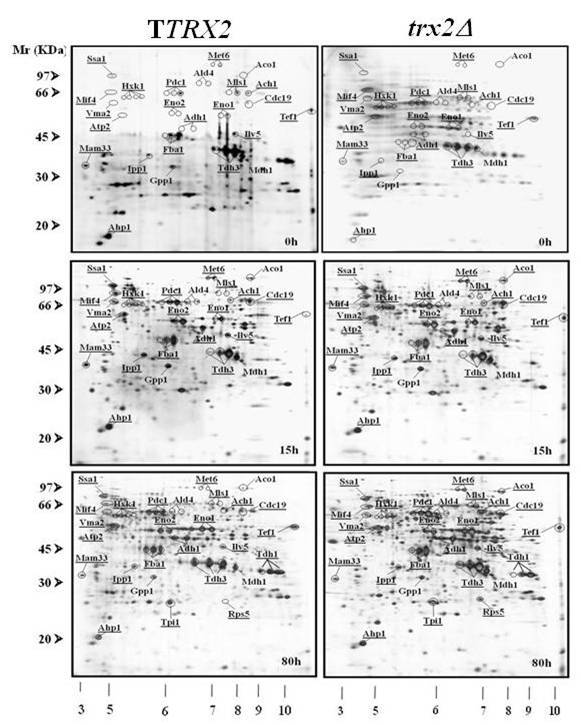

Supplement: Additional file 2 — Two dimensional protein gels during biomass propagation at 0 h, 15 h and 80 h for TRX2 gene modified strains. Proteins were visualized with silver staining and gels were used as loading control. The proteins whose intensity varied significantly (P < 0.05) among the three replicates for each time point were annotated on the gel. [file 1475-2859-11-4-S2.JPEG]

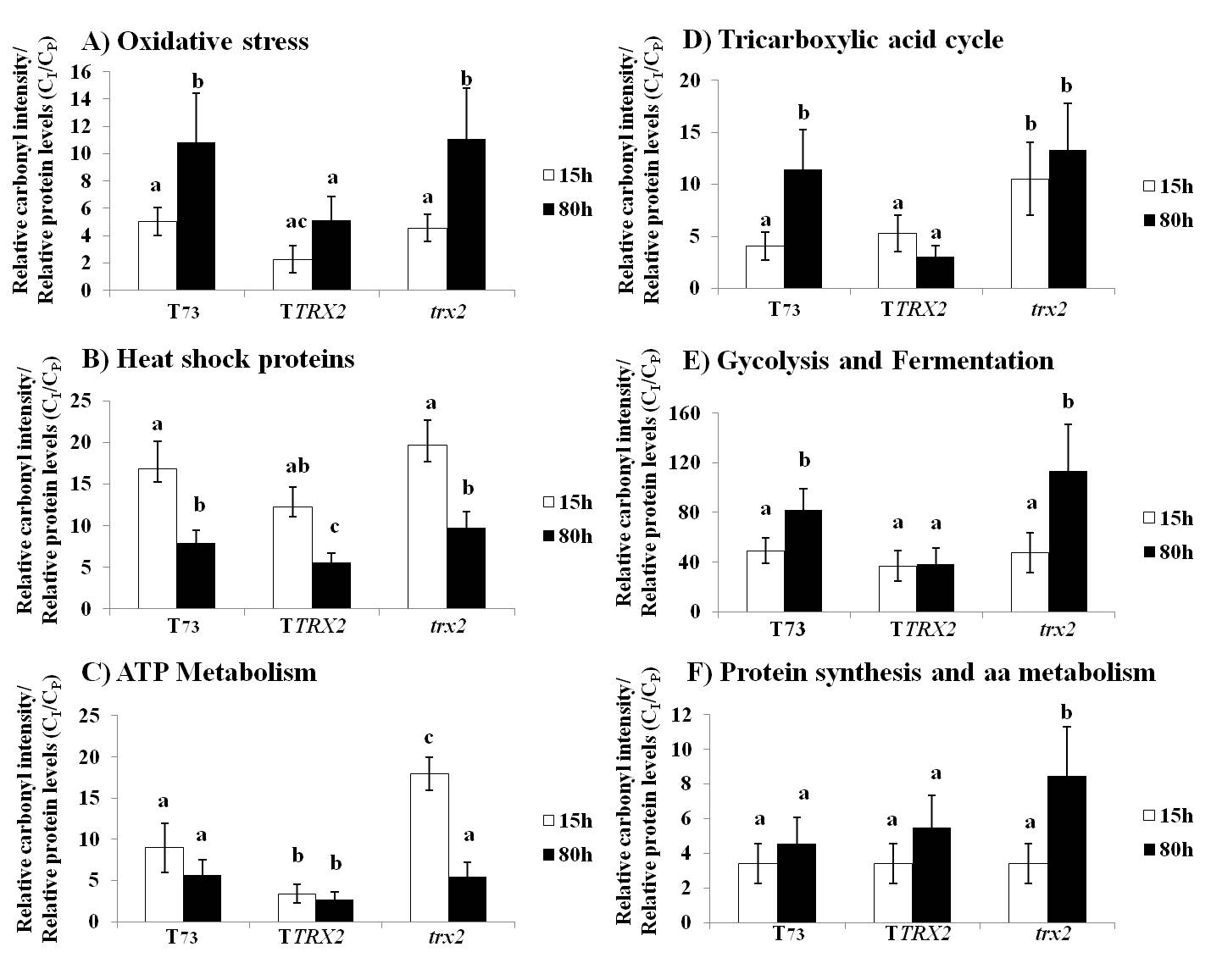

Supplement: Additional file 3 — Carbonylation profile of the different protein functional categories during biomass propagation process. Relative carbonyl content measured as CI/PI of each defined functional category at 15 h and 80 h of growth among the three strains T73, TTRX2 and trx2. All the data are expressed as means ± standard deviation. Comparisons among multiple groups were performed using the ANOVA (a is significantly different (p < 0.05) to b and significantly different to c (p < 0.05). [file 1475-2859-11-4-S3.JPEG]

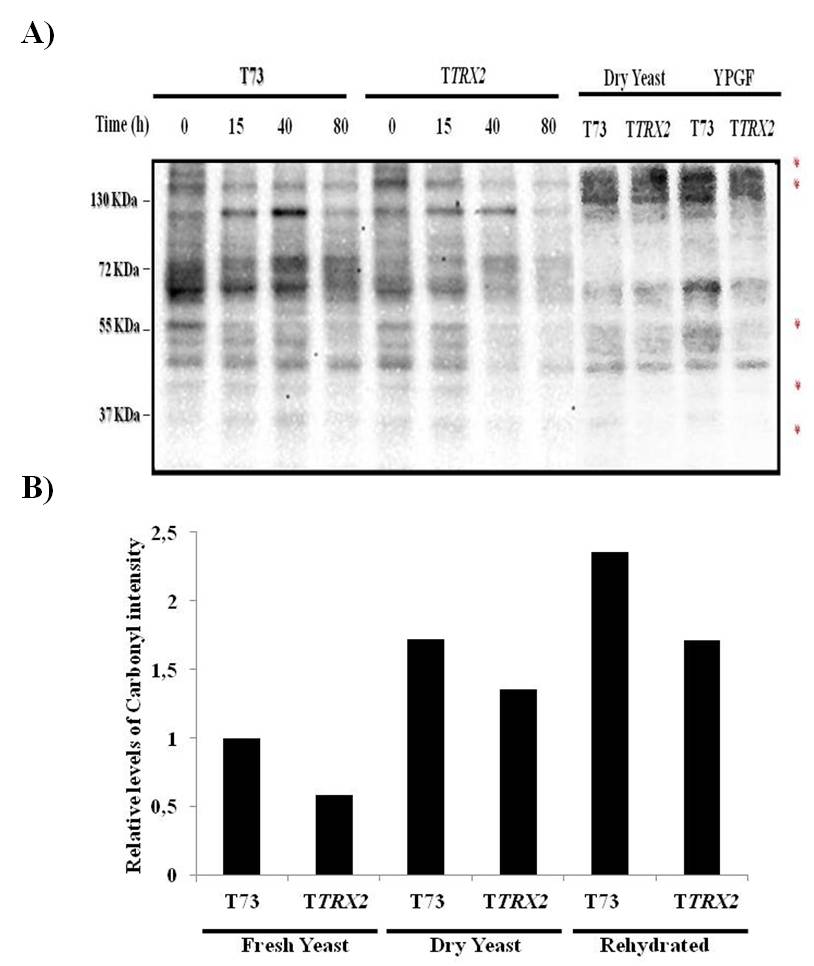

Supplement: Additional file 4 — Carbonyls levels after drying process and YPGF re-inocculation. (A) anti-DNP monodimensional western blot at different time points of the industrial process and after drying and re-inocculation in YPGF for T73 and TTRX2 strains. (B) Relative carbonyl levels were quantified by using the QuantityOne software (Bio-Rad) and carbonylation levels were normalized with the protein amount. [file 1475-2859-11-4-S4.JPEG]

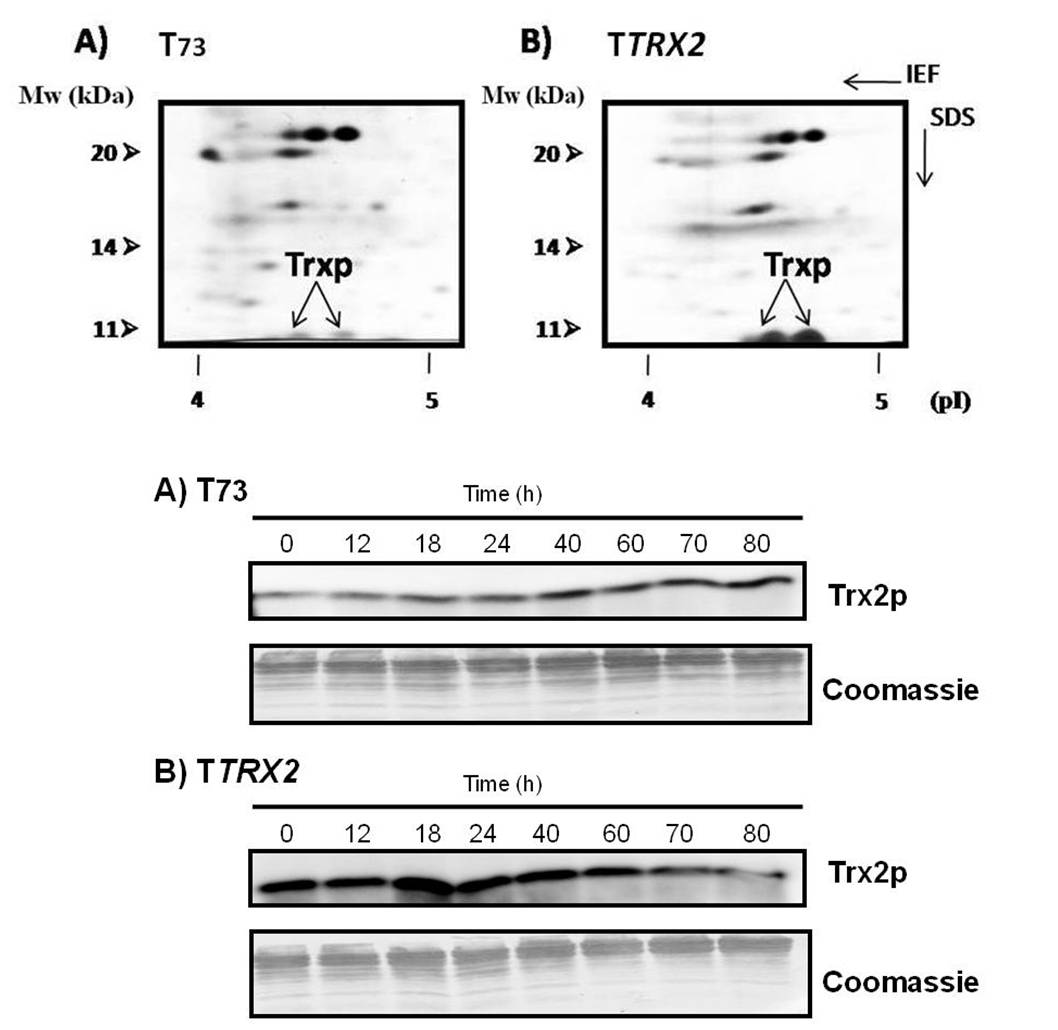

Supplement: Additional file 5 — Trx2p visualization during biomass propagation. (A). Magnified regions of the two-dimensional gels where Trx2p can be observed in the different strains (A) T73 and (B) TTRX2 at 15 h of the biomass propagation process. (B) Western blot anti-Trx2p during biomass propagation. Coomassie-stained membranes are shown as a control of protein amount. [file 1475-2859-11-4-S5.JPEG]
